# Clinical value of radial endobronchial ultrasound combined with metagenomic next-generation sequencing in the malignant tumors patients with pulmonary infection

**DOI:** 10.3389/fcimb.2026.1799148

**Published:** 2026-07-27

**Authors:** Yongchun Song, Xiaojing Zhang, Hongjing Wang, Yufei Wang, Sharan Zhang, Yifan Li, Xinrui Cui, Xiaodan Li, Yuqian Li, Jing Wang, Jingshen Su, Yafeng Zheng, Wei Gai, Wei Liu

**Affiliations:** 1 Department of Radiation Oncology, Tianjin’s Clinical Research Center for Cancer, Key Laboratory of Cancer Prevention and Therapy, Tianjin Medical University Cancer Institute and Hospital, National Clinical Research Center for Cancer, Tianjin, China; 2 WillingMed Technology (Beijing) Co., Ltd, Beijing, China; 3 Tianjin University of Traditional Chinese Medicine, Tianjin, China; 4 Department of Respiratory Medicine, Second Affiliated Hospital of Tianjin University of Traditional Chinese Medicine, Tianjin, China

**Keywords:** BALF, malignant tumors, mNGS, pulmonary infection, R-EBUS

## Abstract

**Introduction:**

Patients treated with systemic anti-tumor therapies are more likely to develop pulmonary infections due to weakened immune systems. This study aims to evaluate the clinical application of radial endobronchial ultrasound (R-EBUS) combined with metagenomic next-generation sequencing (mNGS) in the diagnosis and treatment of pulmonary infections among patients undergoing systemic anti-tumor therapy.

**Methods:**

This study is a single-center retrospective analysis that includes 84 patients with pulmonary infections following systemic anti-tumor therapy. Patients were stratified into sepsis (SOFA score ≥2, n=32) and non-sepsis (SOFA score <2, n=52) groups based on Sepsis-3.0 criteria. BALF samples were subjected to both mNGS and conventional microbiological tests (CMT). Pathogen profiles, diagnostic performance, clinical impact on antimicrobial therapy, and microbiome diversity were analyzed.

**Results:**

mNGS demonstrated a significantly higher positive detection rate than CMT (95.24% vs. 30.95%, *P* < 0.001). mNGS identified a broader spectrum of pathogens, including bacteria, fungi, and viruses, and detected mixed infections more frequently than CMT. The clinical impact of mNGS was positive in 84.52% of cases, primarily by initiating targeted therapy or confirming empirical treatment. Microbiome analysis revealed significantly lower alpha diversity (Shannon, ACE, Chao1 indices) in the severe group compared to the non-severe group.

**Discussion:**

EBUS-guided mNGS of BALF was associated with improved pathogen detection in malignancy patients with pulmonary infections, leading to a high rate of beneficial antimicrobial adjustments. Distinct microbial signatures are associated with infection severity, suggesting potential diagnostic and therapeutic implications.

## 1 Introduction

Patients with malignant tumors are more susceptible to infections due to systemic immunocompromise caused by anticancer therapies (e.g., chemotherapy, radiotherapy, immunotherapy) compared to the general population (Sobhani et al., 2023). Infections increase the risk of ICU admission by about 3.5 times (Kamboj and Sepkowitz, 2009). Acute respiratory infections are responsible for about one-third of infection-related mortality and represent a significant global health and medical burden (Collaborators GLRI, 2018). In patients with malignant tumors, treatment-related immunocompromise increases vulnerability to respiratory infections, and serious infectious complications further worsen prognosis.

Multiple types of respiratory specimens often reflect different airway conditions (Miao et al., 2022). Bronchoalveolar lavage fluid (BALF) obtained via bronchoscopy and saline lavage is commonly used for detecting suspected lower respiratory tract infections, as it provides a comprehensive view of lung condition (Chen et al., 2022). However, the accuracy of pathogen detection can be affected when lesions are located in areas that are difficult to reach with conventional bronchoscopy (Deng et al., 2018). In recent years, radial endobronchial ultrasound (R-EBUS)-guided real-time bronchoalveolar lavage has been applied, particularly for samples that are difficult to obtain using conventional bronchoscopy (Liu et al., 2019). Endobronchial ultrasound (EBUS) offers significant advantages for accurate sampling of deep peripheral lesions, especially in areas such as the hilum and mediastinum, where conventional bronchoscopy is less effective (Biondini et al., 2023). This technique ensures that the BALF collected is from the lesion site, thereby improving the accuracy of pathogen detection.

Early failure to identify pathogens accurately contributes to the high mortality rate in patients with pulmonary infections (Jiang et al., 2024). Clinical microbiology laboratories typically use conventional microbial tests (CMT, e.g., culture, smear microscopy, serology, and PCR) to detect pathogens in samples, but these methods are often time-consuming and lack sensitivity (Wang et al., 2024). For patients with malignant tumors, early and accurate identification of the causative agent is crucial. Metagenomic next-generation sequencing (mNGS), a cutting-edge technology, has been applied to various infectious diseases in recent years. This advanced technique offers high sensitivity, fast turnaround time, and unbiased results, allowing for more accurate identification of infectious agents (Wu et al., 2025). Although several studies have shown that BALF mNGS can effectively detect pathogens in patients with lower respiratory tract infections, data on its application in cancer patients with lung infections undergoing active antineoplastic therapy remain limited (Chen et al., 2022; Gao et al., 2024; Wang et al., 2024).

While the diagnostic value of mNGS has been explored, its specific clinical impact on antimicrobial stewardship and the characteristics of the respiratory microbiome in a cohort of solid malignancy patients with pulmonary infections of varying severity are less well-defined. This study aimed to evaluate the performance of mNGS using EBUS-derived BALF samples, its influence on clinical decision-making, and to characterize the lung microbiome in malignancy patients with pulmonary infections of varying severity.

## 2 Materials and methods

### 2.1 Patients and study design

This retrospective cohort study included 105 patients with malignant tumors and pulmonary infections who were hospitalized in the Department of Respiratory Medicine of the Second Affiliated Hospital of Tianjin University of Traditional Chinese Medicine between July 2023 and September 2025. Inclusion criteria: (1) Age ≥ 18 years; (2) patients with malignant tumors were admitted to the hospital due to pulmonary infection; (3) Prior receipt of systemic therapy against malignant tumors; (4) Patient agrees to use R-EBUS to collect BALF; (5) Complete clinical data available. Exclusion criteria: (1) Patients without malignant tumor; (2) Newly diagnosed malignancy with no prior systemic antineoplastic therapy; (3) Absence of pulmonary infection; (4) Incomplete clinical data. The clinical data of each patient were collected, including demographic characteristics, systemic therapy for malignancy and anti-tumor, laboratory parameters, clinical diagnosis, and treatment details.

BALF samples were used for mNGS detection and culture. Blood, sputum, and other respiratory tract specimens were tested for pathogens by CMT, which included bacterial and fungal cultures, serological antigen testing, and quantitative polymerase chain reaction (PCR) testing for *Klebsiella pneumoniae*, *Haemophilus influenzae*, *Streptococcus pneumoniae*, *Pseudomonas aeruginosa*, respiratory syncytial virus, and influenza A viruses using a respiratory six-panel reagent kit. Clinical judgment was performed by two experienced clinicians.

### 2.2 BALF collection and processing

After obtaining written informed consent, R-EBUS-guided bronchoscopy was performed by an experienced clinician based on the patient’s chest computed tomography (CT) images (Chen et al., 2021). All patients were given general anesthesia when performing EBUS, and the anesthetic drugs were propofol (1 mg/kg) and remifentanil (0.05 μg/kg/min) intravenous anesthesia. The patient’s nasal cavity and pharynx are administered with a local anesthetic with 2% lidocaine, while the patient’s vital signs are measured. Under the guidance of CT images, the ultrafine bronchoscope is extended to the lesion lobe and subsegmental bronchial lumen, the intraluminal ultrasound probe was inserted into the guide sheath, advanced to the target area through the bronchoscopic channel, and ultrasound scanning was initiated upon sensing resistance. Images were monitored until a lesion signal was identified. Once the lesion site is identified, the bronchoscope was secured, the ultrasound probe withdrawn from the guide sheath, and lavage performed along the established path (Li et al., 2020). Subsequently, the extracted specimens are subjected to a Rapid Field Assessment (ROSE). The collected BALF samples were divided into two parts, one sent to WillingMed Technology (Beijing) Co., Ltd for mNGS testing, and the other was immediately sent to the hospital’s microbiology laboratory for conventional microbiological testing.

### 2.3 mNGS detection

Cell-free DNA (cfDNA) from BALF was extracted using the PathoXtract^®^ Basic Pathogen Nucleic Acid Kit (WYXM03211S, WillingMed Corp, Beijing, China) according to the manufacturer’s instructions, and using the PathoXtract^®^ Viral DNA/RNA Isolation Kit (WYXM03009S, WillingMed Corp, Beijing, China) following the manufacturer’s protocol RNA (Li et al., 2022; Li et al., 2024). DNA and RNA were mixed and then the RNA was reverse transcribed into complementary DNA (cDNA) using the SuperScript^®^ Double-Stranded cDNA Synthesis Kit (11917020, Invitrogen). Libraries were prepared using the KAPA DNA HyperPrep Kit (KK8504, KAPA, Kapa Biosystems, Wilmington, MA, United States). Pooled libraries were sequenced on MGISEQ-200 platform using a 50 bp, single-end sequencing kit (MGI Technology) and at least 20 million sequencing reads were acquired for each sample (Yu et al., 2024). Quality control is performed after high-quality sequencing data are obtained. Data consistent with the GRCh37 (hg19) sequence of the human reference genome were screened and removed using Bowtie2 v2.4.3 (Langmead and Salzberg, 2012). Kraken2 v2.1.0 compares the remaining sequences with existing microbial nucleic acid sequences in the database for classification and identification of microorganisms (Wood et al., 2019).

For pathogen identification, a threshold called RPTM (reads per ten million) was used. Bacteria and fungi with RPTM ≥ 20, viruses with RPTM ≥ 3, and special pathogens (including *Cryptococcus* and *Mycobacterium*) with RPTM ≥ 1, were considered as positive (Langelier et al., 2018; Chen et al., 2023). All patients received their mNGS results within 24 hours.

### 2.4 Clinical adjudication of mNGS results and evaluation of impact on treatment

To confirm the composite clinical diagnosis, the clinical committee primarily evaluated it according to standardized criteria for Karius test studies (Blauwkamp et al., 2019; Xu et al., 2023). The clinician panel divided the likelihood of mNGS being a causative agent of a lung infection into four categories: definite, probable, possible, and unlikely. (1) Definite: mNGS results were consistent with CMT results performed within 7 days of mNGS detection; (2) Probable: Microorganisms detected by mNGS could be the cause of infection; (3) Possible: Microorganisms detected by mNGS have the potential to cause infection but were considered uncommon causes by clinical experts based on medical records; (4) Unlikely: Based on other clinical outcomes, the mNGS detection of microorganisms was not identified as a possible cause of infection, or was inconsistent with CMT results. In our study, pathogens classified as definite, probable, and possible were identified as the cause of the patient’s disease, while the unlikely pathogens were classified as colonization or contamination (Xu et al., 2024).

To assess the true impact of mNGS on clinical treatment, two clinical experts retrospectively adjusted antibiotics within 7 days of receipt of the mNGS report, based on mNGS results and with reference to grading criteria (Xu et al., 2023; Feng et al., 2024). The final clinical assessment was divided into three categories: positive, negative, and no impact.

### 2.5 Statistical analysis

All data in this study were statistically analyzed by SPSS 25.0 (USA). Continuous variables are expressed as medians or mean ± standard deviations (SD) with interquartile ranges. Categorical variables are expressed as the number of patients and their proportions. The T-test was used to compare continuous variables, and the chi-square test or Fisher’s exact test was used for categorical variables. *P* < 0.05 is considered statistically significant. We assessed the diagnostic performance of mNGS and CMT for final clinical diagnosis (reference standard) by sensitivity. Sensitivity = true positive/(true positive+ false negative) (Blauwkamp et al., 2019). A multi-faceted comparison of the microbial community structure was conducted in this study. Initially, within-community species richness and evenness were characterized using α-diversity indices, including the Shannon, Simpson, Chao1, and ACE indices. Subsequently, the overall differences in microbial composition between groups were examined by Principal Coordinates Analysis (PCoA) based on the Bray-Curtis distance metric. Furthermore, the Linear Discriminant Analysis Effect Size (LEfSe) method was employed to identify statistically significant discriminatory biomarkers across different groups. All statistical analyses were performed using R software (version 4.5.0) with the aid of packages including Vegan, ape, ggplot2, ggtree, and microeco.

## 3 Results

### 3.1 General characteristics of the patients

**Figure 1:**
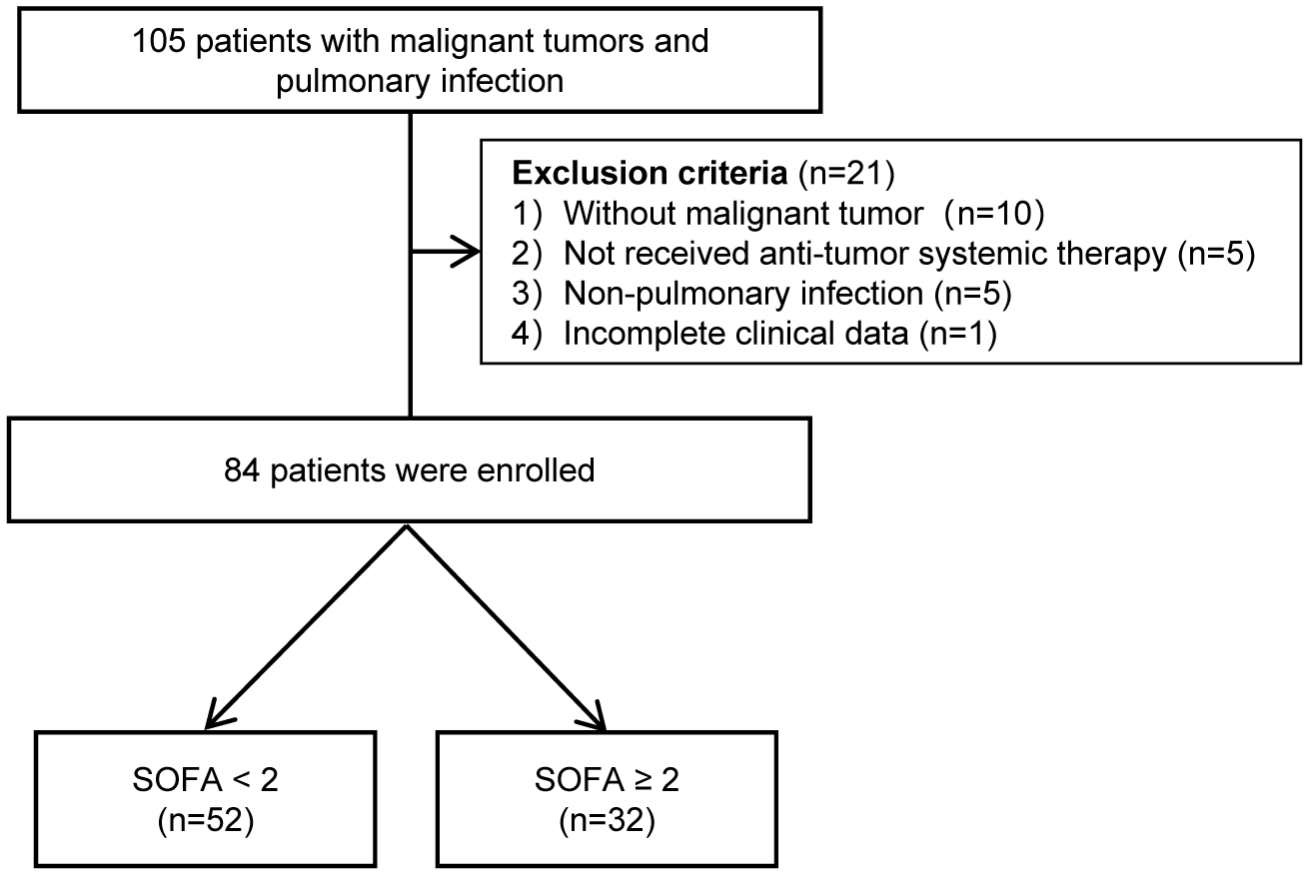
Study flow.

This retrospective cohort study initially screened 105 patients with malignant tumors and suspected pulmonary infections who were hospitalized between July 2023 and September 2025. Of these, 84 patients met the predefined inclusion criteria and were included in the final analysis (**Figure 1**). The baseline characteristics of the study population are summarized in **Table 1**. The mean age was 62.5 ± 8.12 years, and 73.81% were male. Lung cancer was the most common primary malignancy (84.52%), followed by hematological malignancies (9.52%) and other solid tumors (5.95%). The most frequent symptoms were cough (90.48%), breathlessness (61.90%), and fever (57.14%). Based on Sepsis-3.0 criteria, patients were divided into severe (SOFA score ≥2, n=32) and non-severe (SOFA score <2, n=52) groups. There were no significant differences in age, gender, cancer type, or comorbidities between the two groups. However, the sepsis group had a significantly lower proportion of patients who received immunotherapy (34.38% vs. 57.69%, p=0.038) and a significantly lower oxygenation index (262.5 vs. 405, p<0.001).

**Table 1:** Clinical characteristics of 84 enrolled patients.

Characteristics	All (n=84)	Non-severe (n=52)	Severe (n=32)	P-value
Age	62.5 ± 8.12	61.87 ± 8.71	63.53 ± 7.06	0.364
Male, n(%)	62 (73.81)	41 (78.85)	21 (65.62)	0.181
Tumor, n(%)				
Lung	71 (84.52)	44 (84.62)	27 (84.38)	0.976
Esophagus	8 (9.52)	6 (11.54)	2 (6.25)	0.423
Kidney	3 (3.57)	1 (1.92)	2 (6.25)	0.299
Breast	4 (4.76)	2 (3.85)	2 (6.25)	0.615
Ovary	2 (2.38)	2 (3.85)	0 (0.00)	0.262
Thymus	1 (1.19)	1 (1.92)	0 (0.00)	0.43
Lymph	5 (5.95)	3 (5.77)	2 (6.25)	0.928
Bone	4 (4.76)	3 (5.77)	1 (3.12)	0.581
Comorbidities, n(%)				
Hypertension	23 (27.38)	16 (30.77)	7 (21.88)	0.375
Diabetes	15 (17.86)	10 (19.23)	5 (15.62)	0.675
Coronary_heart_disease	17 (20.24)	9 (17.31)	8 (25.00)	0.394
Symptoms, n(%)				
Cough	76 (90.48)	48 (92.31)	28 (87.50)	0.466
Fever	19 (22.62)	11 (21.15)	8 (25.00)	0.682
Breathlessness	52 (61.90)	28 (53.85)	24 (75.00)	0.053
Pleural_effusions	19 (22.62)	11 (21.15)	8 (25.00)	0.682
Tumor treatment, n(%)				
Chemotherapy	63 (75.00)	40 (76.92)	23 (71.88)	0.604
Radiotherapy	55 (65.48)	34 (65.38)	21 (65.62)	0.982
Target	15 (17.86)	9 (17.31)	6 (18.75)	0.867
Immunity	41 (48.81)	30 (57.69)	11 (34.38)	0.038
LOHS	11.71 ± 4.62	11.5 ± 4.22	12.06 ± 5.25	0.591
Clinical index				
White blood cell (10^9/L)	6.63(5.24,8.61)	6.03(5.13,8.11)	6.74(6.07,9.51)	0.25
Neutrophil (%)	77.9 ± 8.43	76.68 ± 7.99	79.88 ± 8.87	0.091
Lymphocyte (%)	13.42 ± 6.06	14.38 ± 5.95	11.88 ± 6.02	0.067
CRP(mg/L)	8.09(4.03,24.65)	6.9(3.39,19.68)	11.65(5.13,34.55)	0.177
PCT(ng/L)	0.05(0.04,0.07)	0.05(0.04,0.07)	0.06(0.04,0.1)	0.355
Red blood cells(×10^12/L)	3.88 ± 0.64	3.92 ± 0.58	3.83 ± 0.72	0.534
Albumin(g/L)	37.1(34.6,39.2)	37.45(34.75,39.7)	36.35(32.18,38.4)	0.131
Platelet (10^9/L)	226.93 ± 80.46	227.85 ± 66.24	225.44 ± 100.56	0.895
Total bilirubin(μmol/L)	6.5(4.7,8.48)	5.85(4.73,9.08)	6.5(4.55,7.63)	0.845
Alanine Transaminase(U/L)	18(11,31)	16.5(11,22.5)	23(13.25,47.75)	0.086
Creatinine(μmol/L)	66.58 ± 16.77	68.91 ± 14.97	62.75 ± 19.01	0.107
Hemoglobin(g/L)	117 ± 16.76	117.42 ± 15.57	116.31 ± 18.76	0.770
PaO2/FiO2(mmHg)	367(284,414)	405(374.5,448.5)	262.5(237.75,294)	<0.001
APACHEII	3.5(2,6)	3(2,5)	4(2.75,6.25)	0.431

CRP, C-reactive protein; PCT, Procalcitonin; SOFA, Sepsis-related organ failure assessment; APACHE II, Acute physiology and chronic health evaluation scoring system.

### 3.2 Pathogen detection performance of mNGS and conventional microbiological tests

**Figure 2:**
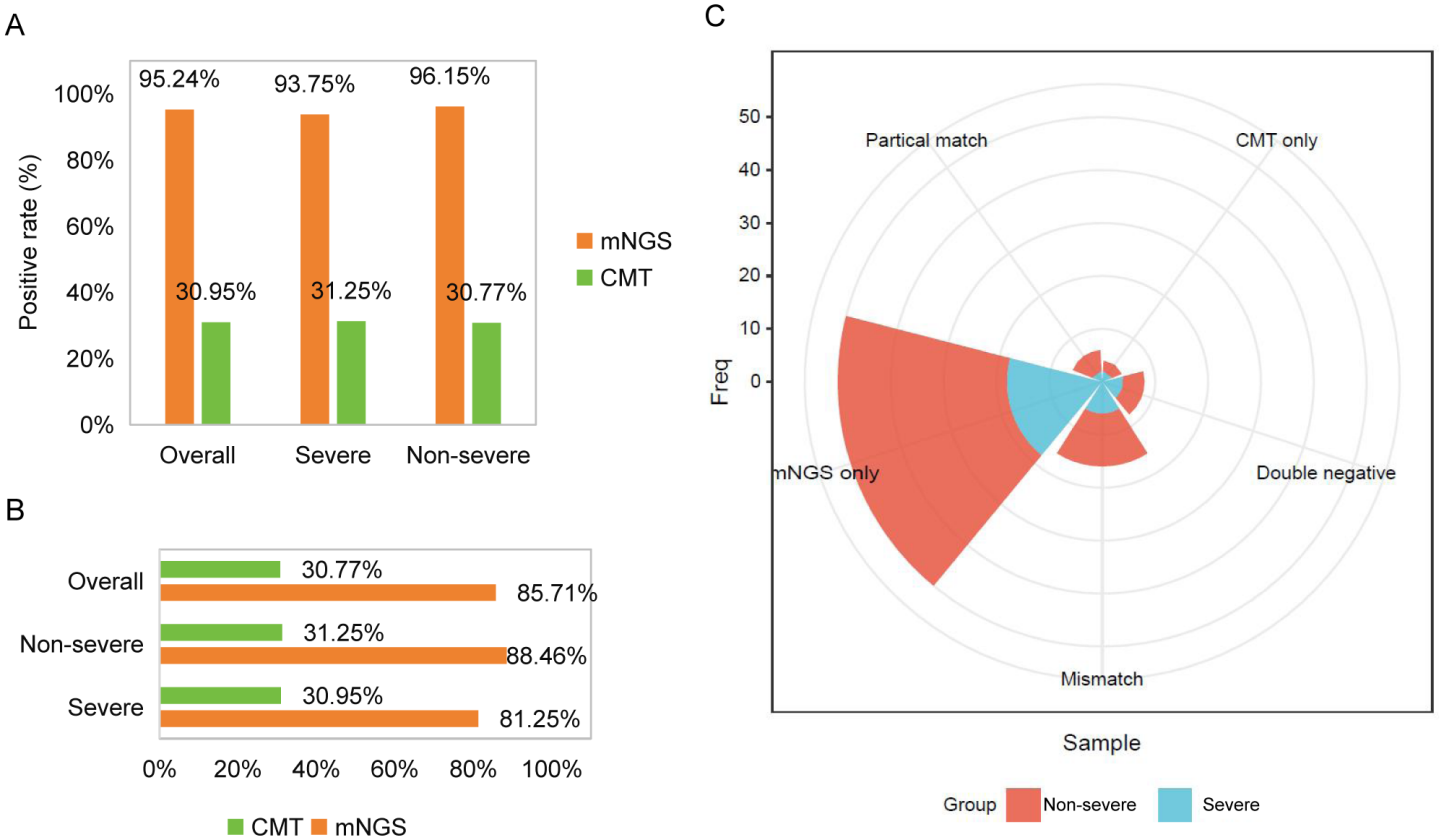
Performance comparison between mNGS and conventional microbial detection. **(A)** Detection positivity rate of mNGS and CMT. **(B)** Detection sensitivity of mNGS and CMT based on the final clinical diagnosis of pulmonary infection. **(C)** Pathogen detection consistency between mNGS and CMT.

The overall pathogen detection performance of mNGS and CMT was compared. mNGS demonstrated a significantly higher positive detection rate compared to CMT (95.24% vs. 30.95%, *P* < 0.001)(**Figure 2A**). Using the final clinial diagnosis as the reference standard, mNGS also demonstrated a significantly higher sensitivity compared to CMT (85.71% vs. 30.77%, *P* < 0.001)(**Figure 2B**). The same trend was observed in the severe (81.25% vs. 30.95%, *P* < 0.001) and non-severe (88.46% vs. 31.25%, *P* < 0.001) groups. Subsequent analysis focused on the pathogen concordance between mNGS and CMT. In both the severe and non-severe groups, the proportion of samples that were positive solely by mNGS was the highest, accounting for 56.25% and 61.54%, respectively. This was followed by the proportion of double-positive samples. In the severe group, double-positive samples constituted 25%, while samples with concordant pathogens accounted for 6.25%. In the non-severe group, double-positive samples represented 26.92%, and those with concordant pathogens accounted for 7.69% (**Figure 2C**).

### 3.3 Pathogen spectrum and distribution

A total of 68 pathogens identified by mNGS were classified as causative pathogens. These comprised 40 bacterial species (20 Gram-positive and 20 Gram-negative), 15 fungal species, 12 viruses, and one atypical pathogen (the *Mycobacterium tuberculosis* complex). In contrast, CMT identified only 7 pathogens to the species level, including 2 Gram-negative bacteria, 1 Gram-positive bacteria, and 4 fungi.

**Figure 3:**
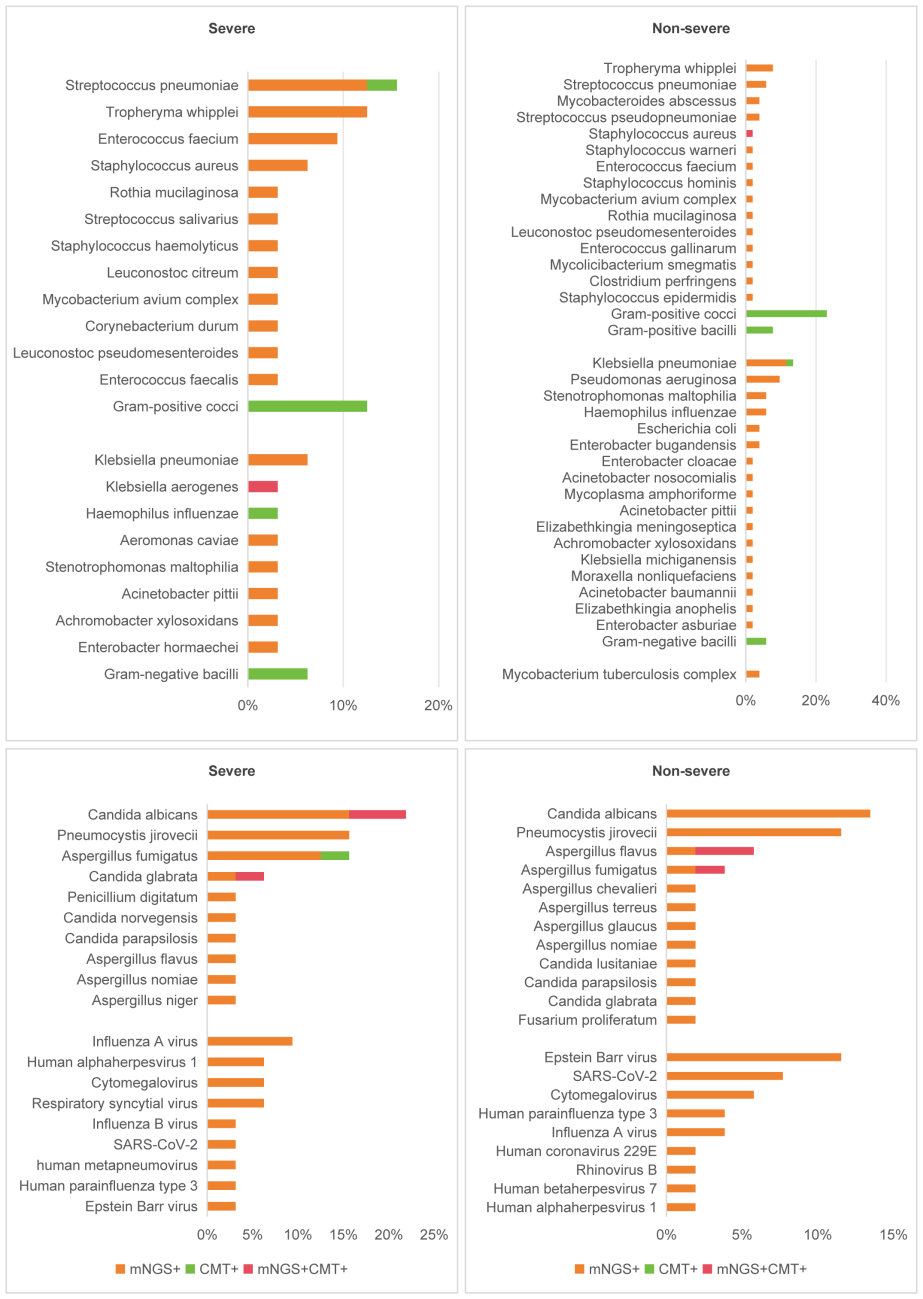
Etiological spectrum of lung infection in patients with malignant tumors.

The most prevalent Gram-positive bacteria in the severe group was *Streptococcus pneumoniae*, followed by *Tropheryma whipplei* and *Enterococcus faecium*. In the non-severe group, *Tropheryma whipplei* was the most common, followed by *Streptococcus pneumoniae*, *Mycobacteroides abscessus*, and *Streptococcus pseudopneumoniae*. For Gram-negative bacteria, *Klebsiella pneumoniae* was slightly more frequent in the severe group. In the non-severe group, the most common were *Klebsiella pneumoniae*, *Pseudomonas aeruginosa*, *Stenotrophomonas maltophilia*, and *Haemophilus influenzae*. The infection rate of Gram-positive bacteria was higher than that of Gram-negative bacteria in the severe group, but this trend was not pronounced in the non-severe group (**Figure 3**).

*Candida albicans* and *Pneumocystis jirovecii* were the most frequently detected fungi in both groups, with a higher fungal detection rate observed in the severe group. In addition, *Aspergillus flavus*, *Aspergillus fumigatus*, *Aspergillus nomiae*, *Candida parapsilosis* and *Candida glabrata* were also identified in both groups. While *Penicillium digitatum*, *Candida norvegensis*, and *Aspergillus niger* were detected exclusively in severe patients and *Aspergillus chevalieri*, *Aspergillus terreus*, *Aspergillus glaucus*, *Candida lusitaniae* and *Fusarium proliferatum* were detected only in non-severe patients. mNGS successfully detected all fungal species present; however, CMT identified only four of them.

Regarding viruses, the most common in the severe group were Influenza A virus, HSV-1, CMV, and RSV; whereas in the non-severe group, EBV, SARS-CoV-2, and CMV were predominant (**Figure 3**).

The distribution of non-causative pathogens detected by mNGS is shown in **Supplementary Figure 1**. Viruses were the most common among these, but some fungi and bacteria were also present, such as *Aspergillus fumigatus*, *Pneumocystis jirovecii*, and *Klebsiella pneumoniae*.

### 3.4 Diagnostic performance of mNGS vs. CMT across different infection types

**Figure 4:**
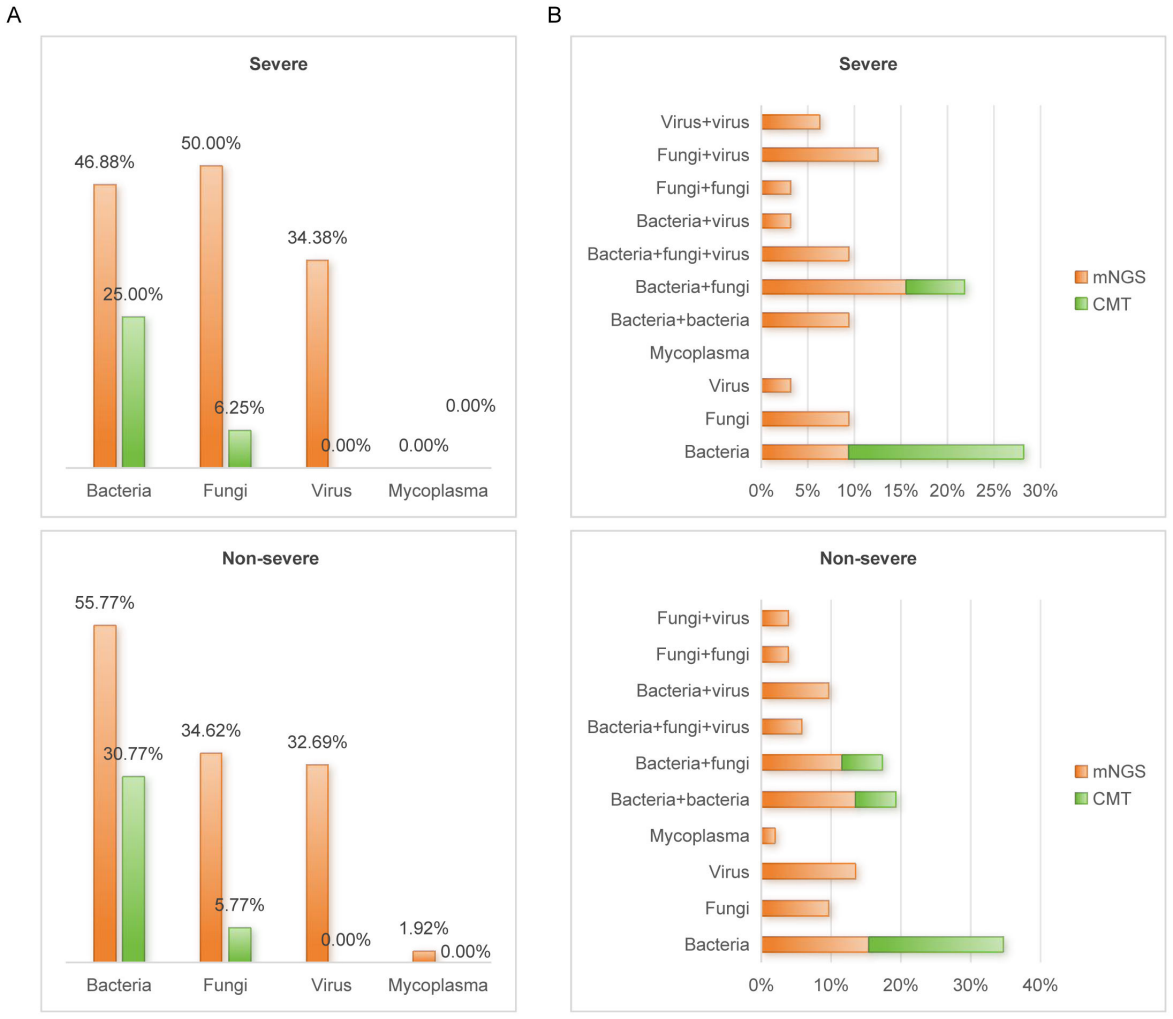
Diagnostic performance of mNGS vs. CMT across different infection types. **(A)** Diagnostic performance of mNGS vs. CMT for bacteria, fungi and virus. **(B)** Diagnostic performance of mNGS vs. CMT for single and mixed infection.

In both non-severe and severe patients, the diagnostic capability of mNGS for bacteria, fungi, viruses, and *Mycoplasma* was superior to that of CMT (**Figure 4A**). Furthermore, compared to CMT, mNGS demonstrated a significant advantage in detecting mixed infections. This advantage was particularly pronounced in severe patients, where mNGS detected mixed infections in 59.38% of cases, compared with 6.25% by CMT (*P* < 0.001). Bacteria were the most commonly detected type of single infection by both methods in both patient groups. Regarding mixed infections, bacterial-bacterial co-infections were most frequent in non-severe patients, followed by bacterial-fungal and bacterial-viral combinations. In severe patients, bacterial-fungal co-infections were the most common, followed by fungal-viral combinations (**Figure 4B**).

### 3.5 Clinical impact of EBUS combined with mNGS on antimicrobial therapy

EBUS combined with mNGS had a significant positive effect on the treatment of patients with pulmonary infections, with positive effects observed in 81.25% of severe patients and 86.54% of non-severe patients. A positive clinical impact was primarily observed in cases where mNGS results led to the initiation of appropriate targeted therapy or confirmed the validity of existing empirical treatment. No impact was mainly associated with positive mNGS detections (11.25%), where the current antibiotic therapy was already effective against the detected pathogens. Notably, no negative clinical impact on antimicrobial management resulted from the mNGS findings (**Table 2**). Among cases with fungal pathogens identified by mNGS, antifungal therapy was initiated in 90% of cases; in the remaining three, the existing antimicrobial regimen already provided adequate coverage.

**Table 2:** Classification of the clinical effects of EBUS combined with mNGS on pulmonary infection in patients with malignant tumors.

Clinical impact	Grade	Description	Overall	Non-severe	Severe
Positive	M1	Initialization of the appropriate antibiotics treatment	38 (45.24%)	24 (46.15%)	14 (43.75%)
	M2	Antibiotic escalation	10 (11.90%)	6 (11.54%)	4 (12.50%)
	M3	Antibiotic de-escalation	1 (1.19%)	1 (1.92%)	0
	M4	Confirmed empirical treatment	22 (26.19%)	14 (26.92%)	8 (25.00%)
No impact	M5	No adjustment in treatment while the result was positive	9 (10.71%)	5 (9.62%)	4 (12.50%)
	M6	The patient was discharged or dead	0	0	0
	M7	No adjustment in treatment while the result was negative	4 (4.76%)	2 (3.85%)	2 (6.25%)
Negative	M8	NGS led to unnecessary treatment	0	0	0

### 3.6 Microbiome analysis

**Figure 5:**
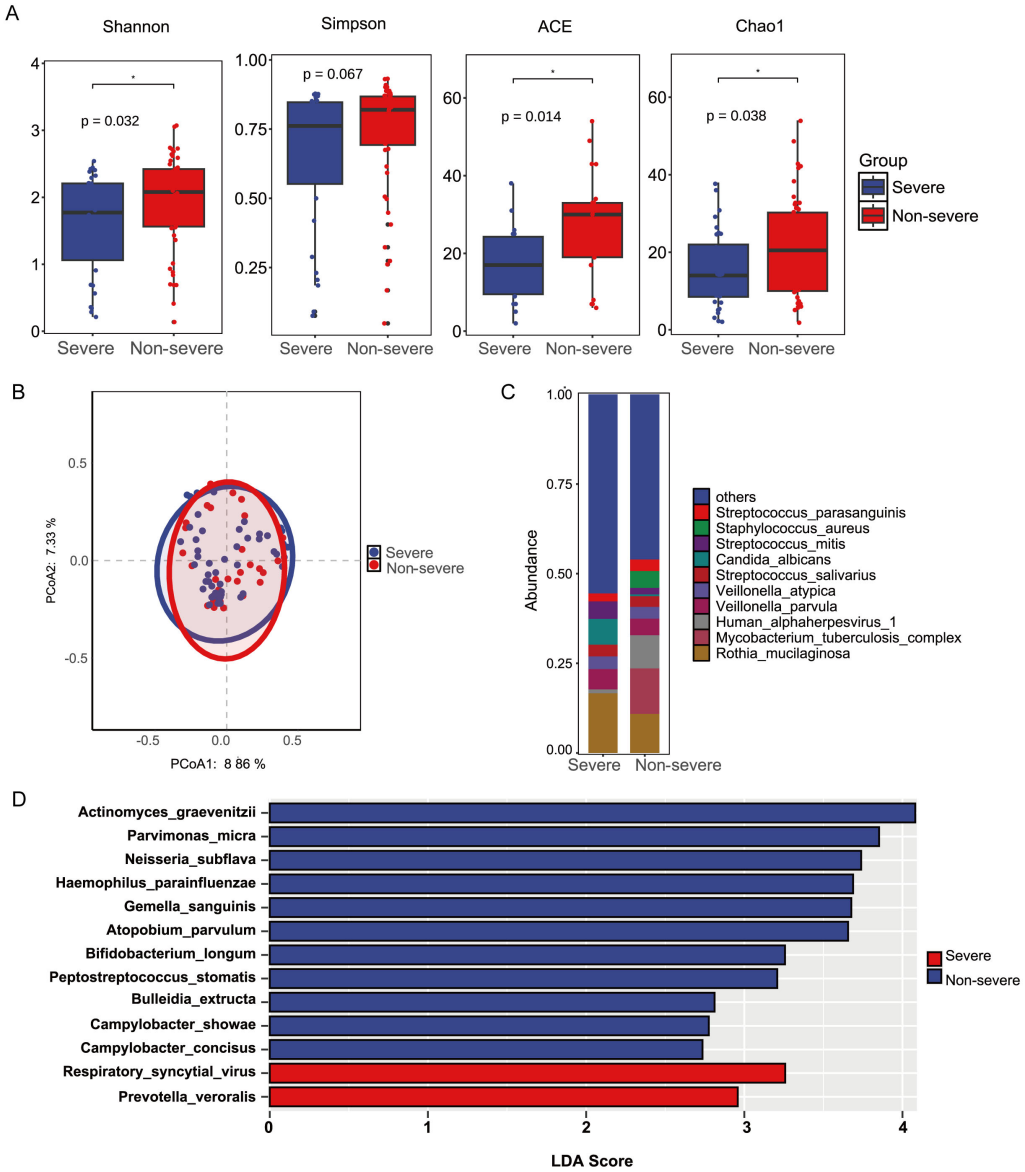
Differences in lung microbial composition between severe and non-severe patients with malignant tumors. **(A)** alpha diversity. **(B)** PCoA result. **(C)** Bar plot showing the ten most abundant species. **(D)** Lefse analysis identifying discriminatory microbial biomarkers between groups.

Microbiome analysis revealed significant differences between the severe and non-severe groups. Alpha diversity, measured by Shannon, ACE, and Chao1 indices, was significantly lower in the severe group (*P* < 0.05), indicating reduced microbial richness and evenness (**Figure 5A**). Beta diversity analysis (PCoA) showed an overlap in microbial community structure between the two groups, suggesting a similar overall microbiota composition (**Figure 5B**). Distinct microbial compositions were observed between the Severe and Non-severe groups. *Rothia mucilaginosa* was the most abundant species in the Severe group, followed by *Candida albicans*. In contrast, the *Mycobacterium tuberculosis complex* and *Rothia mucilaginosa* were predominant in the Non-severe group. Additionally, species such as *Streptococcus mitis*, *Streptococcus salivarius*, and *Veillonella atypica* demonstrated relatively higher abundances in the Severe group (**Figure 5C**). Linear discriminant analysis (LDA) identified specific species as key discriminators between the groups. Respiratory Syncytial Virus (RSV) and *Prevotella veroralis* were enriched in the severe group, while *Actinomyces graevenitzii* and *Parvimonas micra* were more abundant in the non-severe group (**Figure 5D**).

## 4 Discussion

Cancer patients are more likely to develop infections due to factors such as local tumor influence, systemic treatment of cancer, destruction of physical barriers, neutropenia, and cell dysfunction, among which respiratory tract infection is the primary cause (Seo et al., 2021). Poor prognosis is often attributable to delayed or inaccurate pathogen identification. Compared with other types of specimens, BALF is widely used in the detection of lower respiratory tract infections (Xiong et al., 2020; Lin et al., 2022; Xiao et al., 2023). However, for deep lesions that cannot be reached by bronchoscopy, the sensitivity of BALF obtained by conventional bronchoscopy as a pathogen detection sample is often reduced. Over the past two decades, EBUS has emerged as a highly effective and minimally invasive technique for pathological examination of peribronchial and mediastinum, and more recently, EBUS has assisted in the diagnosis of various lung diseases, including malignancies and infections (Jalil et al., 2015). Although many studies have evaluated the performance of BALF mNGS in various lung disease infections, relevant studies remain limited owing to the small and clinically specific population of patients undergoing active anti-tumor therapy. To our knowledge, relatively few studies have simultaneously characterized the full pathogen spectrum and the clinical impact of mNGS-guided antimicrobial stewardship, and the respiratory microbiome signatures associated with infection severity, specifically in cancer patients undergoing active systemic antineoplastic therapy.

The diagnostic superiority of mNGS over CMT observed in our study - with a sensitivity of 85.71% versus 30.95% and an overall positivity rate of 95.24% - is consistent with, yet quantitatively higher than, findings reported in prior series from general clinical populations. In a multicenter retrospective study of 246 patients with suspected pulmonary infection, BALF mNGS demonstrated a sensitivity of 53.49% versus 23.26% for CMT, with particularly pronounced advantages for atypical pathogens (100% vs. 7.14%), viruses (92.31% vs. 7.69%), and fungi (78.57% vs. 39.29%) (Jin et al., 2022). In a larger series of 502 patients with suspected pneumonia across mixed comorbidity groups, mNGS achieved a diagnostic sensitivity of 72.5% and accuracy of 74.9%, outperforming CMT sensitivity of 25.4% (Wu et al., 2022). The higher positivity rate in our cohort may reflect the elevated infectious burden and pathogen complexity inherent to patients receiving active antineoplastic therapy, in whom immunosuppression is more profound and pathogen diversity is broader than in general hospital populations. Consistent with this interpretation, Wang et al. reported an mNGS sensitivity of 100% specifically in cancer patients with severe pneumonia, with antimicrobial treatment optimization achieved in 62.90% of cases following mNGS results (Wang et al., 2024). Importantly, the diagnostic advantage of mNGS was maintained in our cohort despite the fact that 88.1% of patients had received antimicrobial agents prior to sampling — a scenario in which culture-based methods are most severely compromised (Jiang et al., 2024; Lv et al., 2024; Zhang et al., 2024).

Our study illustrates the epidemiological spectrum of lung infections in cancer patients, with bacteria being the most common pathogens, particularly *Streptococcus pneumoniae* and *Klebsiella pneumoniae* (**Figure 3**). This is consistent with a previous meta-analysis of infections in cancer patients (Wang et al., 2023). Furthermore, the infection rate of Gram-positive bacteria was higher than that of Gram-negative bacteria in the severe group, but this trend was not pronounced in the non-severe group. This association between infection type and disease severity can provide more precise guidance for medication. Although *Klebsiella pneumoniae* and *Streptococcus pneumoniae* often colonize the respiratory tract, the high pathogen load reported by mNGS and clinical findings suggest they are clinically identified as the cause of infection. Fungal infections are a significant cause of pulmonary infections, particularly in immunosuppressed cancer patients (Seo et al., 2021). mNGS significantly improves fungal detection rates, with this advantage being more pronounced in the severe group (**Figure 4A**). All fungal pathogens were detected by BALF mNGS, while CMT identified only four species. The most commonly detected fungi were *Candida albicans*, *Pneumocystis jirovecii*, and *Aspergillus fumigatus*, which are also the most common infections in cancer patients in previous studies (Wang et al., 2024). In contrast to prior reports, bacterial-fungal co-infections were the predominant mixed infection pattern in the severe group, and respiratory viruses - including influenza A virus and respiratory syncytial virus - were the most frequently co-detected viral pathogens (Wang et al., 2023). The samples were collected in autumn and winter, and the recent respiratory virus epidemic in northern China may increase patients’ susceptibility to pulmonary infections following anti-tumor therapy. Additionally, repeated hospitalizations of patients with malignant tumors can lead to infections caused by cross-contamination.

The polymicrobial infection rate observed in our cohort (52.38%, 44/84 by mNGS) appears higher than rates reported in general immunocompromised populations, although cross-study comparisons should be interpreted cautiously. A recent study stratified by immune status found mixed infection rates of 35.1% in immunocompromised versus 13.1% in immunocompetent patients, with bacterial-fungal co-infection as the predominant combination and mNGS-guided treatment modifications significantly more frequent in the immunocompromised group (56.8% vs. 35.4%) (Wang et al., 2023). Another large-scale series of 546 patients confirmed that mixed infections were significantly more prevalent in immunocompromised patients (37.8%) compared with immunocompetent counterparts (Liu et al., 2024). The higher polymicrobial rate in our cohort likely reflects the specific immunological consequences of active antineoplastic therapy: Wang et al. specifically identified receipt of anti-tumor therapy as an independent risk factor for mixed infection in cancer patients with severe pneumonia (87.18% vs. 65.22%, *P* = 0.04) (Wang et al., 2024), a finding directionally consistent with our observation. In the severe subgroup, the mixed infection rate reached 59.38%, further supporting the association between infection severity and pathogen complexity in this population (Liu et al., 2024; Li et al., 2026). These findings suggest that empirical monotherapy regimens may be insufficient for a substantial proportion of cancer patients on active treatment, and that comprehensive mNGS-based pathogen characterization is essential for appropriate antimicrobial coverage.

The clinical impact rate observed in our study (84.52%, M1–M7, with M8 = 0) appears higher than that reported in prior series, although differences in study design and patient populations should be considered. One published study reported treatment adjustment in 56.8% of cases in an immunocompromised group (Li et al., 2026), while another study observed antimicrobial optimization in 62.90% of cancer patients with severe pneumonia following mNGS (Wang et al., 2024), and general RICU cohorts have reported clinical impact rates of approximately 56-65% (Lv et al., 2024; Zhang et al., 2024). A multicenter propensity score-matched study further demonstrated that mNGS use was significantly associated with improved 21-day clinical outcomes in immunocompromised patients compared with CMT alone, suggesting a causal pathway from sequencing-guided diagnosis to clinical benefit (Guo et al., 2026). The particularly elevated clinical impact rate in our cohort may be attributable to the high prevalence of CMT-invisible pathogens: Tropheryma whipplei (8 cases), non-tuberculous mycobacteria (5 cases), and rare fungal species (26 cases) -organisms undetectable by any conventional microbiological method - collectively accounted for approximately 27% of the total cohort and in every case required mNGS-exclusive identification to guide appropriate therapy. The fungal detection rate in our cohort (40.48% overall; 40.63% in the severe group) was consistent with but somewhat lower than that reported for immunocompromised patients in general (43.2%) (Li et al., 2026), possibly reflecting the heterogeneous composition of the malignancy-related immunosuppression in our cohort compared with populations defined by single immune-depleting conditions. Taken together, these comparisons suggest that the clinical impact of EBUS-guided BALF mNGS may be greater in cancer patients on active antineoplastic therapy relative to general immunocompromised populations, driven by a distinct pathogen ecology that disproportionately favors organisms beyond the detection capacity of conventional diagnostics (Lin et al., 2022; Wang et al., 2024; Guo et al., 2026; Li et al., 2026).

The microbiome analysis provides novel insights. The significantly reduced alpha diversity in the severe group supports the concept that a loss of microbial community stability and complexity is associated with a more severe disease state, a phenomenon observed in other critical illnesses (Wypych et al., 2019; King, 2024). Differences in beta diversity were observed between groups, suggesting that the lung microbiome composition may be associated with disease severity. The identification of specific discriminatory taxa - including enrichment of *Actinomyces graevenitzii* in non-severe cases, and the RSV in severe cases, points toward potential microbial biomarkers of disease severity. This echoes research in other fields where specific microbiota signatures have been linked to clinical outcomes (Cheng et al., 2024).

Several limitations of this study warrant consideration. First, as a single-center retrospective design with a relatively small sample size, selection bias and limited generalizability cannot be excluded, and large-scale prospective multicenter validation is needed. Second, distinguishing true infection from colonization remains inherently challenging with mNGS; its high sensitivity increases detection of respiratory colonizers and non-viable pathogen fragments, and fixed RPTM thresholds - though widely adopted - lack universal standardization across specimen types and patient populations, with pathogen-specific threshold optimization showing meaningful diagnostic improvement in recent studies (Li et al., 2025). Third, the relatively high cost of mNGS currently limits routine adoption, and formal cost-effectiveness analyses specific to oncology patients with pulmonary infection remain lacking (Osei Sekyere, 2025). Fourth, mNGS provides limited real-time capacity for antimicrobial resistance gene interpretation, as genotype-to-phenotype prediction remains an unresolved translational challenge (Elbehiry and Abalkhail, 2025). Finally, in the 46.43% of our patients who received immunotherapy, differentiating infectious pneumonia from immune checkpoint inhibitor–related pneumonitis represents a diagnostic complexity not fully addressed by the current study design, and future work should incorporate prospective multidisciplinary adjudication in this subgroup (Jiang et al., 2026). Future studies should prioritize prospective multicenter cohorts with standardized mNGS thresholds validated for immunocompromised cancer patients, and explore the integration of host transcriptomic profiling with pathogen detection (Kalantar and Langelier, 2021).

In conclusion, EBUS-guided BALF mNGS was associated with improved etiological diagnosis of pulmonary infections in malignancy patients, leading to impactful changes in antimicrobial therapy. The distinct respiratory microbiome associated with infection severity suggests that microbial ecology may play a role in disease progression. Prospective, multicenter studies are needed to validate these findings and to explore the potential of microbiome-based diagnostics and therapeutics in this vulnerable population.

## Data Availability

The datasets presented in this study can be found in online repositories. The names of the repository/repositories and accession number(s) can be found below: https://www.ncbi.nlm.nih.gov/, PRJNA1204144.
