# Supplementary material for: Clinical value of radial endobronchial ultrasound combined with metagenomic next-generation sequencing in the malignant tumors patients with pulmonary infection
Source: Front Cell Infect Microbiol. 2026 Jul 27;16:1799148. doi: 10.3389/fcimb.2026.1799148 (PMC13454537; doi:10.3389/fcimb.2026.1799148)
Supplement: Supplementary file 1 [file Supplementaryfile1.docx]

Supplementary Material

# Supplementary Figures

**Supplementary Figure 1.** The distribution of non-causative pathogens detected by mNGS.
